# Quantitative Archaeological Feature Identification Using Handheld Spectrometers

**DOI:** 10.3390/s26102935

**Published:** 2026-05-07

**Authors:** Yoon Jung Choi

**Affiliations:** 1 Daeyang Humanity College, Sejong University, 209 Neungdong-ro, Gwangjin-gu, Seoul 05006, Republic of Korea; yoonchoi@sejong.ac.kr; 2 Department of Energy Resources and Geosystems Engineering, Sejong University, 209 Neungdong-ro, Gwangjin-gu, Seoul 05006, Republic of Korea

**Keywords:** soil spectroscopy, feature identification, VIS-NIR spectroscopy

## Abstract

Soil colour and texture play important roles in identifying archaeological features during excavations, particularly in rescue archaeology where rapid and reliable interpretation is required. This study investigated the application of visible-near-infrared (VIS-NIR) soil spectroscopy for quantitatively characterising cultural heritage materials and archaeological soils on freshly exposed surfaces after topsoil removal during excavation. Surface soil spectra were collected using a portable spectrometer from nine features at a rescue excavation site in Hyeondo-myeon, Republic of Korea. A PCA-based spectral deviation approach was applied to detect deviations of archaeological soils from locally defined natural background spectra. Balanced accuracy values exceeded 0.70 under optimised configurations across all sites, with several sites achieving values above 0.80. Strong statistical discrimination coincided with spatially coherent clustering of elevated anomaly values corresponding to archaeologically identified feature zones. The 400–1000 nm wavelength range, combined with locally calibrated background spectra, yielded the most stable and reproducible performance. The proposed workflow demonstrates that field-based VIS-NIR spectroscopy can provide rapid, quantitative, and spatially interpretable support for archaeological feature identification. By integrating sensor-based spectral characterisation with anomaly mapping, the approach minimises interpretive subjectivity and improves analytical reproducibility in excavation decision-making processes.

## 1. Introduction

During archaeological excavation, overburden is removed to expose fresh, undisturbed surfaces on which archaeological features can be identified [1,2]. This stage, commonly referred to as feature identification [3], is critical for determining excavation strategy and documentation. Archaeologists distinguish features such as pits, ditches, graves, and structural remains based on differences in soil colour [4,5,6,7,8,9,10,11] and texture [12,13,14,15,16] relative to surrounding background soils [1,17,18,19,20,21,22]. However, this identification process depends on individual experience and professional judgement [1,23] and therefore can be subjective. As a result, subtle archaeological traces may be overlooked, and natural soil variations may be misinterpreted as anthropogenic features.

Although scientific methods such as geophysical applications [24,25,26,27,28] and remote sensing [29,30,31,32,33] have significantly improved the detection of buried archaeological remains, these technologies are primarily applied prior to excavation and provide limited support during the actual excavation stage. Similarly, conventional soil chemical analyses, which can yield valuable information about settlements and anthropogenic activities, mainly rely on laboratory-based wet chemistry, which is time-consuming and impractical under field conditions [34]. Therefore, despite advances in scientific techniques, feature identification during excavation still relies heavily on individual inspection and personal experience. In rescue archaeology, where time constraints limit repeated verification, the absence of a systematic and reproducible analytical framework can increase the risk of misinterpretation. Therefore, a rapid, quantitative, and field-applicable method for objective feature identification is needed.

Efforts to reduce subjectivity have included standardised colour recording using the Munsell Soil Colour Chart [35,36,37]. However, visual assessment remains sensitive to lighting conditions, observer variability, and soil moisture [38,39,40,41]. Micromorphological analysis and other laboratory-based techniques can provide detailed information on anthropogenic deposits [42] but are unsuitable for rapid field application due to their analytical time requirements. Spatial documentation methods such as GIS and photogrammetry have improved recording accuracy [1,43]. However, these methods do not directly provide quantitative material characterisation for feature differentiation.

Considering that soil colour and texture reflect underlying physical and chemical properties, these characteristics can be quantified through spectral analysis. Spectroscopy extends observations into non-visible wavelengths and enables reproducible quantitative measurements. Accordingly, visible-near-infrared (VIS-NIR) spectroscopy has been widely used in soil science to characterise soil composition and properties [44,45,46,47,48,49,50,51]. Variations in organic matter content, mineral composition, moisture conditions, and anthropogenic inputs can produce measurable spectral differences [49,52,53,54,55,56,57]. Spectral analysis has demonstrated potential for detecting soil and archaeological anomalies [32,58,59]. Previous studies have applied VIS-NIR or infrared spectroscopy to distinguish archaeological deposits from background soils, evaluate sediment layers, and interpret stratigraphic variations [60,61,62,63,64,65,66,67,68]. For example, Choi et al. [60] used a field VIS-NIR spectrometer to statistically distinguish soil reflectance signals and identify archaeological signatures. Haburahj et al. [61] tested whether quantitative colour and spectral measurements can serve as rapid and cost-efficient methods for delineating sediment layers during excavation. Eckmeier [62] applied visible spectroscopy to archaeologically dated pit fills and compared soil colour expressed in terms of luminance and chromaticity to distinguish fills from background sediments. Monnier [63] reviewed the use of infrared spectroscopy in microarchaeological sediments, including methodological issues and applications to organic and inorganic materials. While these studies demonstrate the potential of spectral approaches for analysing archaeological soils and sediments, their applications have largely focused on characterisation and interpretation. Consequently, the use of spectroscopy as a systematic and quantitative decision-support tool during excavation remains underexplored.

Other non-destructive techniques, such as hyperspectral imaging [69,70], X-ray fluorescence (XRF) [71,72], and Raman spectroscopy [73], also offer strong potential for archaeological investigations. These methods can provide detailed compositional or spatial information, but often require specialised instrumentation, controlled acquisition conditions, or complex data processing, which may limit their applicability during active excavation. In contrast, portable VIS–NIR spectroscopy offers a relatively rapid and field-applicable means of capturing localised soil variability during excavation.

In this study, we evaluated the potential of field-based VIS-NIR spectroscopy for quantitative characterisation of archaeological materials during excavation. Building upon previous work, this study developed a structured and statistically evaluated workflow for excavation-stage feature identification using handheld VIS-NIR spectroscopy. By integrating a principal component analysis (PCA) based spectral deviation approach [60] with quantitative performance metrics, we systematically assessed the reliability of spectral differentiation between archaeological materials and surrounding natural soils. The proposed workflow is designed for operation with portable spectrometers under field conditions, providing a practical and reproducible support tool for excavation-stage decision making.

## 2. Materials and Methods

This study evaluated whether surface spectral information can distinguish archaeological features from background subsoil under field conditions. Spectral measurements were collected at a rescue excavation site in the Republic of Korea using a handheld spectrometer.

### 2.1. Study Area

**Figure 1:**
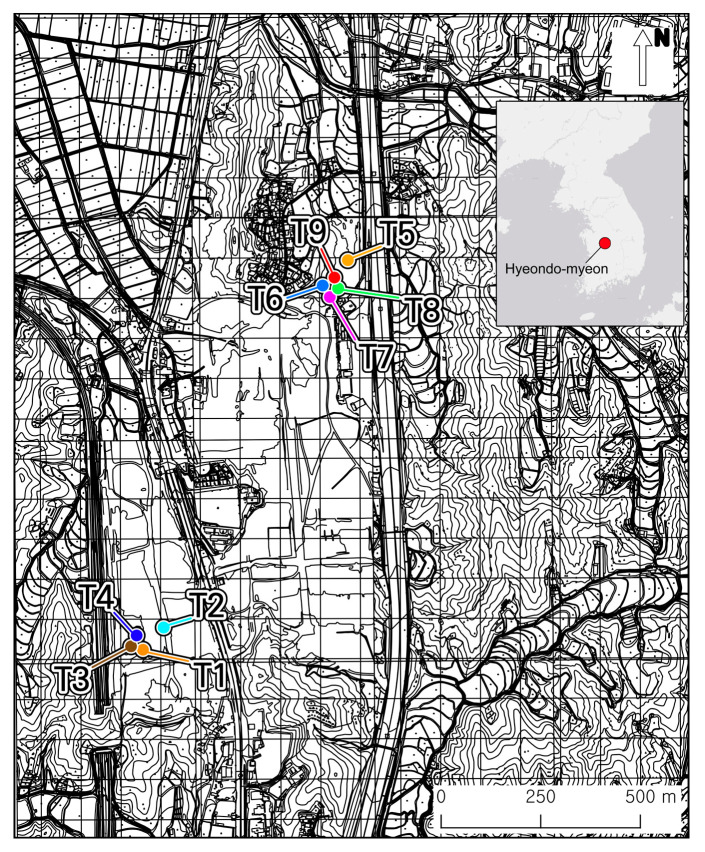
Location of the study area in Hyeondo-myeon, Cheongju-si, Republic of Korea. The nine investigated archaeological features are labelled T1–T9.

The measurements were taken in Hyeondo-myeon, Cheongju-si, Republic of Korea, as shown in Figure 1. Cheongju-si is located in the central region of the country, with a broad intermontane valley between the Charyeong and Noryeong Mountain ranges. The northeastern part of the region consists of moderately elevated terrain (approximately 100 m above sea level), and the topography gradually flattens toward the east, forming a large alluvial terrace. Thus, the region provides favourable conditions for human settlement and has been occupied since the prehistoric period.

**Figure 2:**
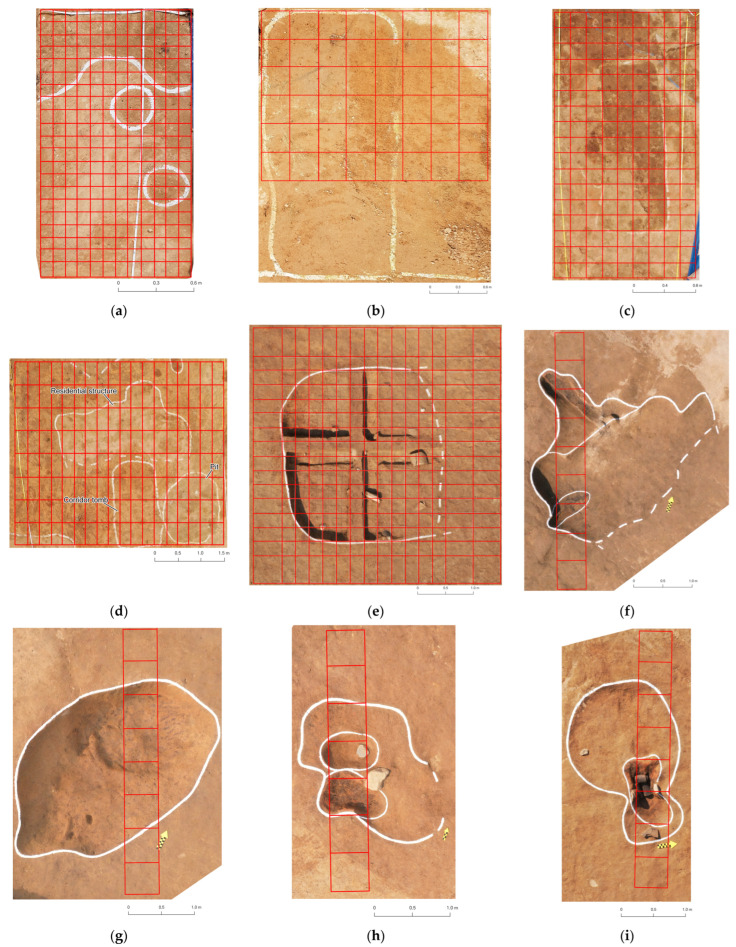
Photographs of the nine investigated excavation surfaces with measurement grids. Due to limited access to the study site, the photographs were acquired at different stages of excavation, whereas all spectra were collected prior to excavation. The boundaries of the archaeological features are outlined in white, and the measurement grids are shown in red. Yellow north arrows are provided in some photographs for orientation. (**a**) Site T1, residential feature from the Joseon Dynasty (AD 1392–1897); (**b**) Site T2, natural feature initially misinterpreted as a tomb; (**c**) Site T3, corridor tomb from the Joseon Dynasty; (**d**) Site T4, mixed feature including a residential structure, corridor tomb, and pit from the Joseon Dynasty; (**e**) Site T5, residential feature from the Joseon Dynasty; (**f**) Site T6, semi-subterranean house from the Joseon Dynasty; (**g**) Site T7, Bronze Age pit; (**h**) Site T8, Joseon Dynasty pit; (**i**) Site T9, Joseon Dynasty pit.

Hyeondo-myeon was under rescue excavation from March 2020 to June 2024, and spectral measurements were taken during the summers of 2020 and 2021. Figure 2 shows photographs of the nine archaeological features where spectral measurements were taken. The archaeological remains identified in the region are mainly residential and burial features, which are generally associated with slash-and-burn farmers from the Joseon dynasty (AD 1392–1897). Here, soil spectra from nine archaeological features, which were primarily identified by archaeologists through traditional feature identification, were carefully measured together with the background subsoil.

Because measurements were conducted during a rescue excavation, access to the study area was limited. Therefore, measurement conditions were not identical for all nine sites (sites T1–T9). For sites T1–T5, the measurement area covered the entire archaeological feature. However, for sites T6–T9, only a transect passing through the feature was measured.

All spectra were collected from exposed surfaces prior to excavation. However, due to field access limitations, the photographs shown in Figure 2 were acquired at different stages of excavation. Sites T1–T4 were photographed immediately after topsoil removal, whereas sites T5–T9 were photographed at later stages of excavation. These differences in photographic timing do not affect the spectral measurements used in this study.

The investigated sites represent a range of archaeological feature types, including residential, tomb-related, and pit features (Table 1). These different contexts may influence soil properties in distinct ways due to variations in formation processes, such as soil mixing, deposition, and localised disturbance, which can result in different spectral responses. A summary of each site, including the size of the measurement area, grid size, and number of spectra per grid, is provided in Table 1. In addition, 166 soil spectra were randomly collected around the study area. Since the study focused on within-site deviation analysis rather than predictive modelling, each spectrum was evaluated individually, and no global classifier was applied. Therefore, spatial sampling imbalance is unlikely to systematically bias the results.

One notable case was site T2, which was a natural feature that was misinterpreted as a tomb during the feature identification process.

### 2.2. Field Spectra Collection

VIS-NIR reflectance of the soil samples was measured using the Analytical Spectral Devices (ASD) TerraSpec Halo Mineral Identifier spectrometer (Malvern Panalytical Ltd., Malvern, UK), which covers wavelengths in the range of 350–2500 nm. The instrument is equipped with a built-in halogen light source and a contact probe that samples reflected light from an area of approximately 1 cm in diameter on the soil surface. The spectral sampling interval was 1 nm, with spectral resolution of 3 nm at 700 nm, 9.8 nm at 1400 nm, and 8.1 nm at 2100 nm. The spectra were calibrated using a white reference panel every 30 measurements.

Spectra were collected from cleaned surfaces immediately after removing topsoil. Measurements were collected from both archaeological features and the surrounding background soils. Figure 2 shows photographs of each archaeological feature and illustrates schematic grid lines of how measurements were taken for each feature. At each grid location, spectra were collected one to three times at randomly selected points within the grid and then averaged to reduce measurement noise.

### 2.3. PCA-Based Spectral Deviation Approach

To identify archaeological materials during feature identification, this study followed the PCA-based spectral deviation approach [60]. The method was formulated as a site-specific anomaly detection framework where PCA was applied only to a group of natural soils (N_soil_) to obtain the spectral characteristic of the natural soils. Based on the principal component (PC) value derived from N_soil_, the measured spectra were recalculated so that each spectrum reflected the characteristic features of N_soil_. The difference between the original spectrum and its reconstructed spectrum derived from N_soil_ was calculated. If the difference was small, the original spectrum was considered to share the characteristics of natural soil. On the other hand, if the difference was large, the original spectrum was considered more likely to represent archaeological soil.

The calculation procedure for this method is described as follows. Principal components derived from the selected N_soil_ dataset (NPCs) were first determined to establish a reference spectral subspace. Using this reference, all spectra, including both archaeological and natural soils, were subsequently reconstructed.

According to the PCA calculation, a spectrum is calculated as shown in Equation (1),

(1) $$S-S_{m}=T_{1}\;*\;PC_{1}+T_{2}\;*\;PC_{2}+residuals\;$$

where *S* is the data (spectrum), *S_m_* is the mean of the dataset (spectra collected), and *T* represents the PC scores. This equation can be rearranged to reconstruct the spectrum of interest *S* using NPC values instead of its original PC scores, producing a reconstructed spectrum *S*′, as shown in Equation (2). In Equation (2), *λ* denotes wavelength and *n* is the number of PCs included in the calculation. Reconstruction was performed using the first *n* number of NPCs only, ensuring that the reconstructed spectra reflected the spectral characteristics of natural soils.

(2) $$S^{\prime }=S_{m}+\sum_{\lambda }^{n}T_{n}\;*\;NPC_{n}\;\;$$

**Figure 3:**
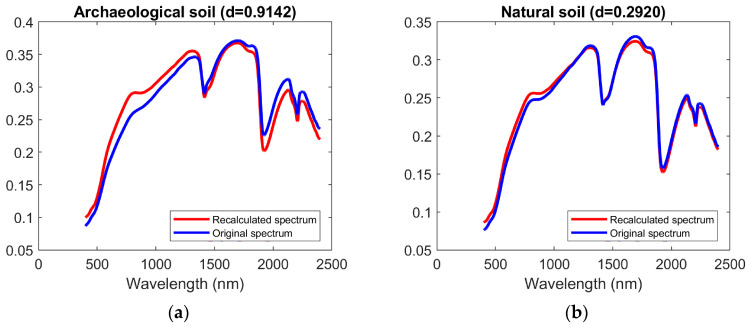
Reflectance spectra of randomly selected archaeological and natural soils before and after proposed PCA-based reconstruction. X- and Y-axis represent wavelength (nm) and reflectance, respectively. The original spectrum (*S*) is shown in blue, and the reconstructed spectrum (*S*′) derived from the proposed method is shown in red. The spectral deviation (*D* value) between *S* and *S*′ is 0.9142 for the archaeological soil and 0.2920 for the natural soil. (**a**) Archaeological soil; (**b**) natural soil.

The Euclidean difference, *D* value, between the original spectrum *S* and reconstructed spectrum *S*′ across the selected wavelength range was calculated to investigate how differently the spectrum behaved compared to N_soil_. Figure 3 illustrates this using randomly selected archaeological and natural soils and shows that both *D* values and spectral shapes differ substantially between archaeological soils and in natural soils after applying the method.

Since there is no predefined threshold, the *D* value was normalised by *D_nat_*, defined as the mean *D* values calculated from all N_soil_ spectra within each site, to obtain the *R* value as shown in Equation (3). Thus, an *R* value greater than or equal to 1 indicated a difference in spectral behaviour from typical natural soil variability, suggesting that the spectrum is more likely influenced by anthropogenic processes. The *R*-value, therefore, represents the spectral deviation from the reference natural soil dataset (N_soil_). These *R* values were calculated for every spectral measurement made within each site.

(3) $$R=\frac{D}{D_{nat}}\;$$

Based on this formulation, the factors influencing the result of this method were (1) spectral preprocessing, (2) wavelength range, (3) N_soil_ selection, and (4) the number of PCs included in the reconstruction.

#### 2.3.1. Spectral Preprocessing

Prior to analysis, pre-treatment was performed to remove the noisy features in the spectra. First, noisy edge regions (below 400 nm and above 2400 nm) were removed, and the whole spectral range was smoothed using a Gaussian convolution with a 10 nm bandwidth to reduce instrumental noise. After smoothing, the spectra were preprocessed using four different pipelines: (1) raw reflectance, (2) standard normal variate (SNV), (3) continuum removal (CR), and (4) first derivative (DER1).

These preprocessing methods were applied to evaluate which spectral characteristics most strongly influenced differences between archaeological and natural soils. Raw reflectance preserves broad colour and albedo contrasts and represents the most basic spectral information. SNV [74] reduces illumination and scatter effects arising from uneven surface roughness, variable compaction, probe-surface contact differences, and small illumination variations during field measurements. CR [75] and DER1 [76] emphasise absorption features and spectral shape rather than overall brightness.

#### 2.3.2. Wavelength Selection

Multiple wavelength ranges were tested to identify the most suitable spectral range for distinguishing archaeological materials. The spectral ranges examined were 400–700 nm (visible), 400–1000 nm (VIS-NIR range [77,78]), 1000–2400 nm (NIR), and 400–2400 nm (full range). In addition to these four primary spectral windows, additional sub-ranges (700–1000, 1000–1800, and 1800–2400 nm) were included to further examine the relative contribution of specific spectral regions. In total, seven spectral windows were evaluated: 400–700, 400–1000, 400–2400, 700–1000, 1000–1800, 1000–2400, and 1800–2400 nm. All analyses were conducted independently for each preprocessing and wavelength combination.

#### 2.3.3. Natural Soil (N_soil_) Selection

In this study, the term ‘natural soil’ refers to all soils which are not part of the archaeological features. The proposed PCA-based method statistically distinguishes soil spectra with anomalous features from the collected reference natural soil spectra, which are referred to as N_soil_.

Three N_soil_ groups were evaluated in this study: local, regional and global. Local N_soil_ refers to natural soils collected within the same site (around each feature), regional N_soil_ includes natural soils collected across the entire study area, and global N_soil_ consists of soil spectra from the ICRAF-ISRIC world soil spectral library [79]. The ICRAF-ISRIC spectral library consists of 4437 samples from 58 countries, where the samples were collected using an ASD FieldSpec FR (Malvern Panalytical Ltd., Malvern, UK) spectrometer (350–2500 nm) under laboratory conditions. For the global N_soil_ dataset, all soil spectra included in the analysis were resampled to a 10 nm resolution, as spectra provided from the spectral library were originally resampled at 10 nm intervals.

**Figure 4:**
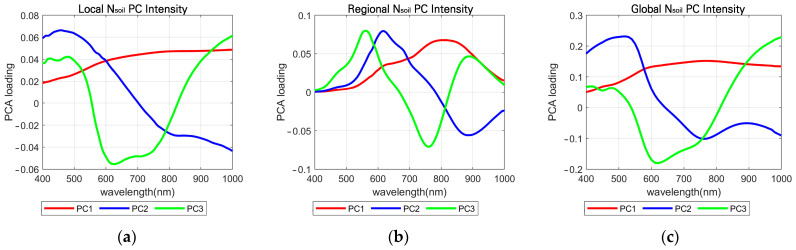
Principal component (PC) loading plots used for N_soil_ selection. PC1, PC2, and PC3 are shown in red, blue, and green, respectively. (**a**) Local N_soil_ loading plot, derived from natural soil spectra collected around each measurement site; (**b**) regional N_soil_ loading plot, derived from natural soil spectra collected across all study sites; (**c**) global N_soil_ loading plot, derived from soil spectra obtained from the ISRIC online spectral library.

Figure 4 shows the first three PC spectra of the three different N_soil_ groups, and Table 2 presents the variance of the first three PCs. All three N_soil_ groups show distinct spectral characteristics in their first three PCs.

**Table 2:** Variance of the first three principal components for groups of soil spectra gathered from each site and the variance of the first three principal components of three groups of N_soil_ (local, regional, and global).

Site	Variance of PC1 (%)	Variance of PC2 (%)	Variance of PC3 (%)
T1	96.3	2.5	0.8
T2	99.4	0.3	0.2
T3	97.3	1.6	0.7
T4	94.2	3.5	1.7
T5	98.9	0.6	0.4
T6	97.5	1.5	0.9
T7	95.4	3.6	0.7
T8	97.3	2.0	0.6
T9	93.4	4.9	1.1
Local N_soil_	98.8	1.0	1.5
Regional N_soil_	99.9	0.01	0.01
Global N_soil_	93.4	3.9	2.3

#### 2.3.4. PC Set

For most soil spectra, the first three PCs typically account for approximately 75% of the variation in the data [55]. The soil spectra collected in Hyeondo-myeon showed that over 95% of the variance was captured by the first three components. More specifically, the first PC alone explained more than 90% of the variation at all sites, as shown in Table 2. Table 2 presents the variance distribution of PCs for all nine study sites. This result suggests that the first PC may be sufficient for analysis.

However, the study investigated up to three PCs to assess whether additional information from higher-order components contributed to improved discrimination. Thus, the optimal number of PCs for identifying soil spectral differences was also systematically evaluated.

### 2.4. Evaluation Strategy

Based on the proposed PCA-based calculation, the study evaluated the optimal conditions for identifying archaeological materials during the feature identification process using VIS-NIR soil spectra data. The evaluated parameters included the preprocessing pipeline, different wavelength ranges, N_soil_ definition, and the number of PCs used. To assess the discriminative capability of these factors, three complementary metrics were employed: balanced accuracy, effect size (Cohen’s d), and the Mann–Whitney U test.

Balanced accuracy [80] was used as the primary performance metric, as it accounts for class imbalance between archaeological and natural soils. It represents the mean of sensitivity and specificity, where sensitivity corresponds to the correct identification of archaeological materials and specificity represents the correct rejection of natural soils. This metric, therefore, provided an intuitive measure of how effectively the method distinguished archaeological features from the background soil.

Effect size [81], quantified using Cohen’s d, measures the separation between archaeological and natural soil R values in standard deviation units. Typically, d values greater than 0.8 indicate strong separation. This metric was used to quantify the magnitude of spectral differentiation, providing a measure of archaeological interpretability beyond simple classification accuracy.

Finally, the Mann–Whitney U test [82] was applied to assess the statistical significance of observed differences without assuming a normal distribution. This approach was particularly suitable for this study, as soil spectral distributions are rarely normal, and sample sizes per site were relatively small.

The combined use of these three metrics enabled robust identification of optimal analytical conditions while reducing the risk of overinterpreting statistically weak or archaeologically insignificant separations. Because this study did not aim to build a predictive classifier but rather to evaluate spectral separability, classification metrics were used only to quantify discrimination performance under a fixed threshold (*R* ≥ 1).

## 3. Results

### 3.1. PCA of the Spectral Dataset

Across all nine study sites, the first PC (PC1) accounted for more than 90% of the total spectral variance (Table 2), indicating that a dominant spectral trend explained most of the variability.

**Figure 5:**
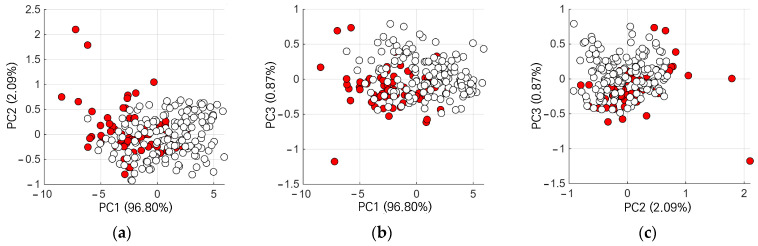
Principal component analysis (PCA) score plots for site T1. Archaeological soils are shown as red markers and natural soils as white markers. (**a**) PC1–PC2 score plot; (**b**) PC1–PC3 score plot; (**c**) PC2–PC3 score plot.

As shown in Figure 5, PCA score plots did not exhibit clear separation between archaeological and natural soil spectra. However, directional tendencies along PC1 were observed for most sites. Although this pattern was not uniformly observed across all datasets, comparable patterns appeared in multiple cases. Figure 5 shows the PCA score plot for a selected study site, site T1. Here, the archaeological soil spectra (red markers) were distributed toward negative PC1 values, whereas natural soils (white markers) were more frequently distributed in the positive PC1 region.

These observations indicate that although PCA score plots did not provide clear separation, deviation from the natural soil reference space captured spectral differences between archaeological and natural soils.

### 3.2. Site-Specific Classification Performance: Optimal Condition

#### 3.2.1. Overall Performance Observation

The proposed PCA-based method quantified spectral differences between archaeological material and natural soils and produced meaningful discrimination performance across the nine study sites. Table 3 summarises the optimal analytical conditions for each site, including wavelength range, N_soil_ definition, preprocessing pipeline, and number of PCs used in the proposed PCA-based calculation, together with balanced accuracy, effect size (Cohen’s d), and Mann–Whitney U test results.

**Figure 6:**
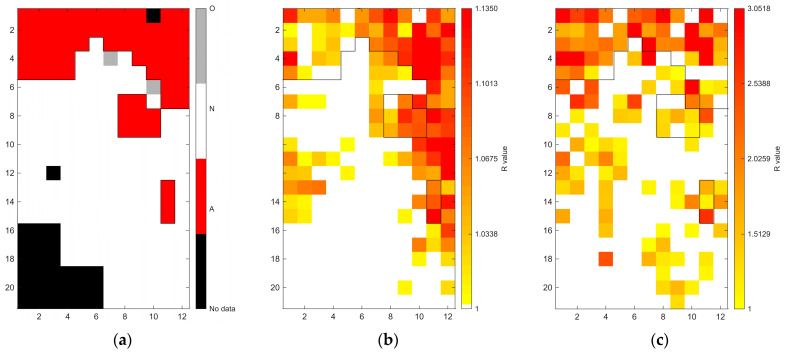
Two-dimensional (2D) distribution of *R* values for site T1. X- and Y-axis represent relative grid positions and do not correspond to actual spatial dimensions. (**a**) Reference classification map showing archaeological soils (red), natural soils (white), unmeasured grid cells (black), and other materials (gray; e.g., paint or modern disturbance during excavation). (**b**) *R* value map under the site-specific optimal configuration (400–1000 nm wavelength range, regional N_soil_, raw spectra, and PC1 reconstruction). Cells with *R* ≥ 1 are displayed using a yellow-to-red gradient, with increasing intensity corresponding to higher *R* values; those with *R* < 1 are shown in white. (**c**) *R* value map under the cross-site standardized configuration (400–1000 nm wavelength range and local N_soil_), using the best-performing preprocessing and PC selection within this constraint.

**Figure 7:**
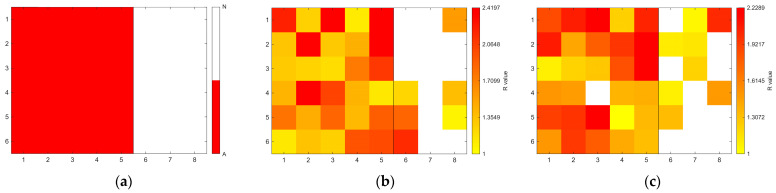
Two-dimensional (2D) distribution of *R* values for site T2. X- and Y-axis represent relative grid positions and do not correspond to actual spatial dimensions. (**a**) Reference classification map showing archaeological (red) and natural (white) soils. (**b**) *R* value map under the site-specific optimal configuration (400–2400 nm wavelength range, local N_soil_, CR spectra, and PC1-3 reconstruction). Cells with *R* ≥ 1 are displayed using a yellow-to-red gradient, with increasing intensity corresponding to higher *R* values; those with *R* < 1 are shown in white. (**c**) *R* value map under the cross-site standardised configuration (400–1000 nm wavelength range and local N_soil_), using the best-performing preprocessing and PC selection within this constraint.

**Figure 8:**
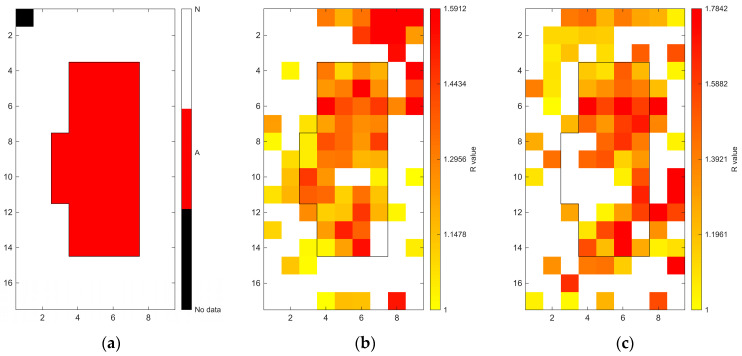
Two-dimensional (2D) distribution of *R* values for site T3. X- and Y-axis represent relative grid positions and do not correspond to actual spatial dimensions. (**a**) Reference classification map showing archaeological (red) and natural (white) soils. (**b**) *R* value map under the site-specific optimal configuration (1000–2400 nm wavelength range, regional N_soil_, SNV-preprocessed spectra, and PC1–3 reconstruction). Cells with *R* ≥ 1 are displayed using a yellow-to-red gradient, with increasing intensity corresponding to higher *R* values; those with *R* < 1 are shown in white. (**c**) *R* value map under the cross-site standardised configuration (400–1000 nm wavelength range and local N_soil_), using the best-performing preprocessing and PC selection within this constraint.

**Figure 9:**
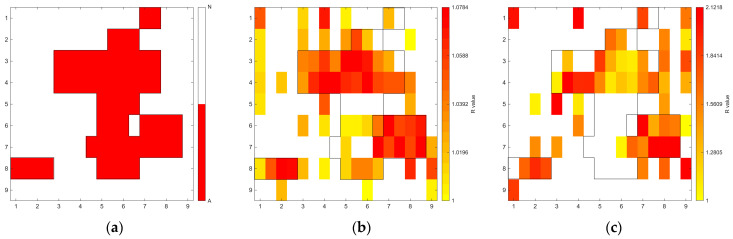
Two-dimensional (2D) distribution of *R* values for site T4. X- and Y-axis represent relative grid positions and do not correspond to actual spatial dimensions. (**a**) Reference classification map showing archaeological (red) and natural (white) soils. (**b**) *R* value map under the site-specific optimal configuration (700–1000 nm wavelength range, global N_soil_, CR spectra, and PC1-3 reconstruction). Cells with *R* ≥ 1 are displayed using a yellow-to-red gradient, with increasing intensity corresponding to higher *R* values; those with *R* < 1 are shown in white. (**c**) *R* value map under the cross-site standardised configuration (400–1000 nm wavelength range and local N_soil_), using the best-performing preprocessing and PC selection within this constraint.

**Figure 10:**
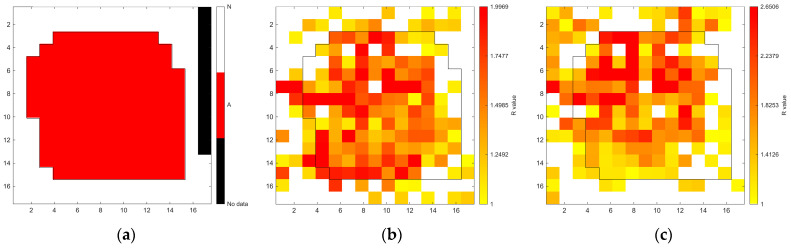
Two-dimensional (2D) distribution of *R* values for site T5. X- and Y-axis represent relative grid positions and do not correspond to actual spatial dimensions. (**a**) Reference classification map showing archaeological (red) and natural (white) soils. (**b**) *R* value map under the site-specific optimal configuration (400–1000 nm wavelength range, regional N_soil_, SNV-preprocessed spectra, and PC1 reconstruction). Cells with *R* ≥ 1 are displayed using a yellow-to-red gradient, with increasing intensity corresponding to higher *R* values; those with *R* < 1 are shown in white. (**c**) *R* value map under the cross-site standardised configuration (400–1000 nm wavelength range and local N_soil_), using the best-performing preprocessing and PC selection within this constraint.

**Figure 11:**
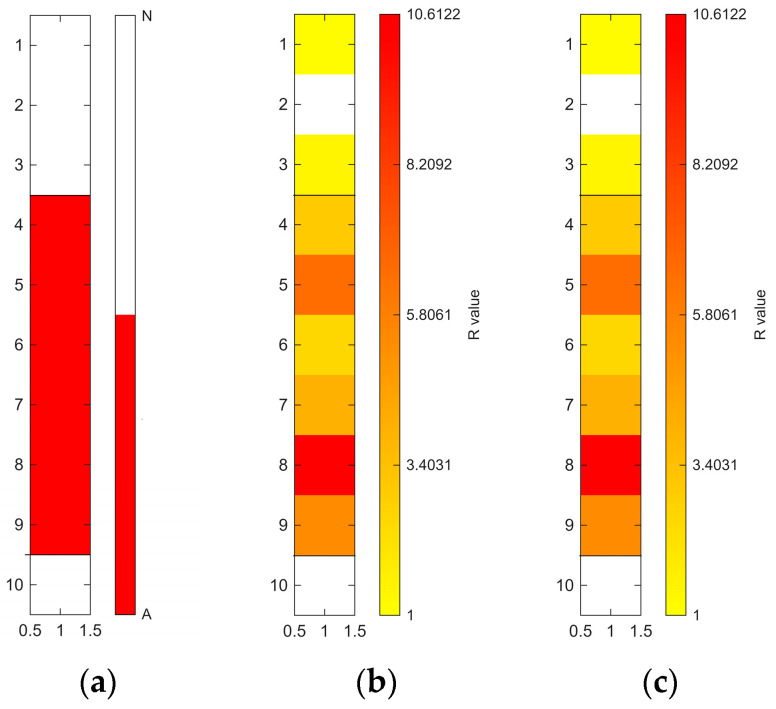
Two-dimensional (2D) distribution of *R* values for site T6. X- and Y-axis represent relative grid positions and do not correspond to actual spatial dimensions. (**a**) Reference classification map showing archaeological (red) and natural (white) soils. (**b**) *R* value map under the site-specific optimal configuration (400–1000 nm wavelength range, local N_soil_, raw spectra, and PC1-3 reconstruction). Cells with *R* ≥ 1 are displayed using a yellow-to-red gradient, with increasing intensity corresponding to higher *R* values; those with *R* < 1 are shown in white. (**c**) *R* value map under the cross-site standardised configuration (400–1000 nm wavelength range and local N_soil_), using the best-performing preprocessing and PC selection within this constraint.

**Figure 12:**
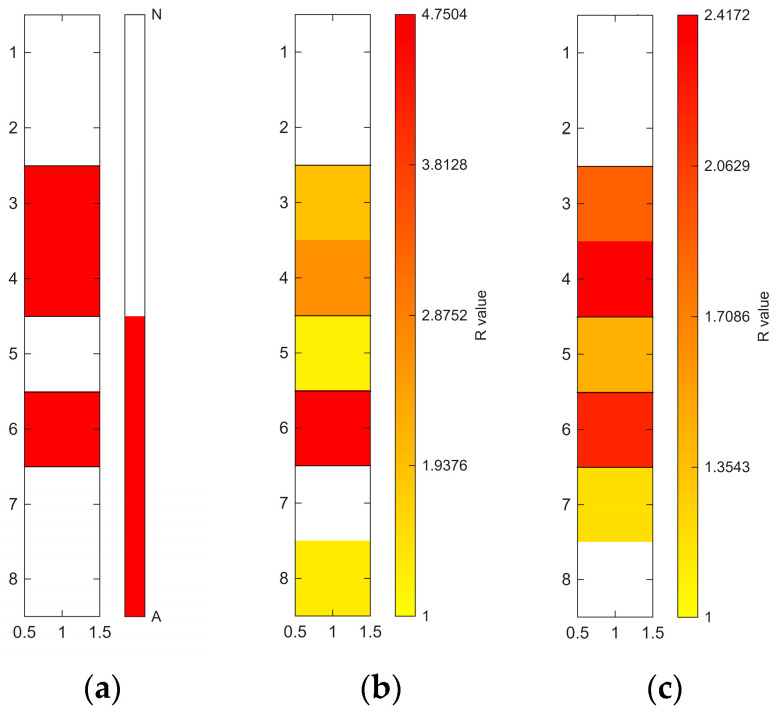
Two-dimensional (2D) distribution of *R* values for site T7. X- and Y-axis represent relative grid positions and do not correspond to actual spatial dimensions. (**a**) Reference classification map showing archaeological (red) and natural (white) soils. (**b**) *R* value map under the site-specific optimal configuration (400–700 nm wavelength range, local N_soil_, raw spectra, and PC1-3 reconstruction). Cells with *R* ≥ 1 are displayed using a yellow-to-red gradient, with increasing intensity corresponding to higher *R* values; those with *R* < 1 are shown in white. (**c**) *R* value map under the cross-site standardised configuration (400–1000 nm wavelength range and local N_soil_), using the best-performing preprocessing and PC selection within this constraint.

**Figure 13:**
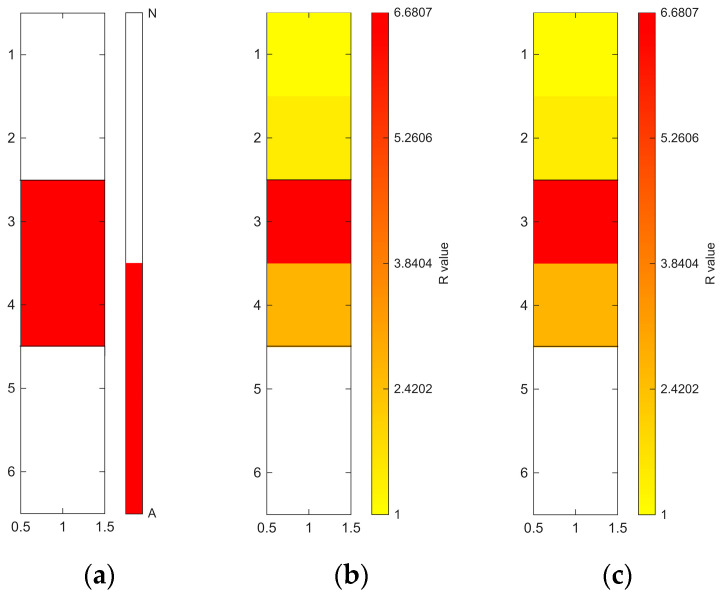
Two-dimensional (2D) distribution of *R* values for site T8. X- and Y-axis represent relative grid positions and do not correspond to actual spatial dimensions. (**a**) Reference classification map showing archaeological (red) and natural (white) soils. (**b**) *R* value map under the site-specific optimal configuration (400–1000 nm wavelength range, local N_soil_, CR spectra, and PC1–3 reconstruction). Cells with *R* ≥ 1 are displayed using a yellow-to-red gradient, with increasing intensity corresponding to higher *R* values; those with *R* < 1 are shown in white. (**c**) *R* value map under the cross-site standardised configuration (400–1000 nm wavelength range and local N_soil_), using the best-performing preprocessing and PC selection within this constraint.

**Figure 14:**
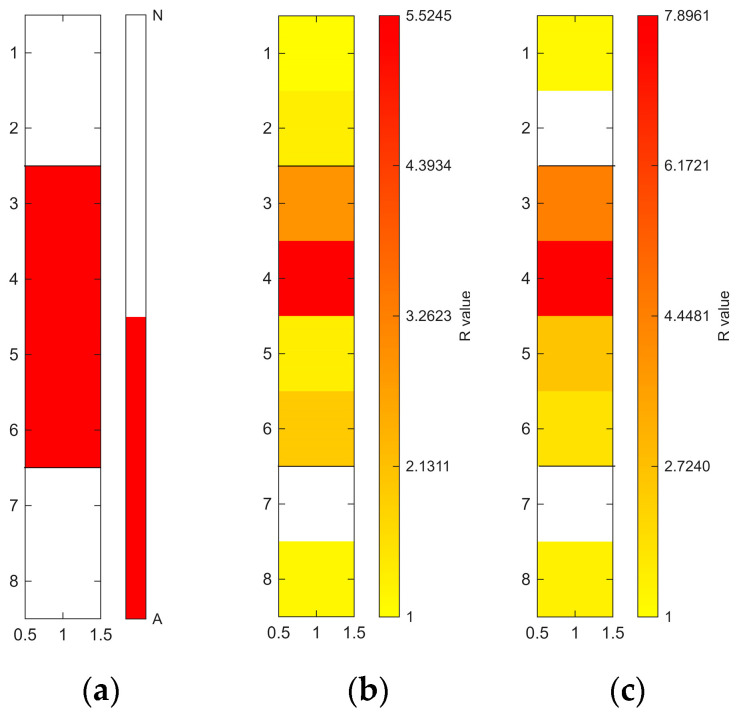
Two-dimensional (2D) distribution of *R* values for site T9. X- and Y-axis represent relative grid positions and do not correspond to actual spatial dimensions. (**a**) Reference classification map showing archaeological (red) and natural (white) soils. (**b**) *R* value map under the site-specific optimal configuration (1000–1800 nm wavelength range, local N_soil_, raw spectra, and PC1–3 reconstruction). Cells with *R* ≥ 1 are displayed using a yellow-to-red gradient, with increasing intensity corresponding to higher *R* values; those with *R* < 1 are shown in white. (**c**) *R* value map under the cross-site standardised configuration (400–1000 nm wavelength range and local N_soil_), using the best-performing preprocessing and PC selection within this constraint.

Figure 6, Figure 7, Figure 8, Figure 9, Figure 10, Figure 11, Figure 12, Figure 13 and Figure 14 present the spatial distribution of *R* values for sites T1–T9, respectively. In each figure, panel (a) shows the reference map based on archaeological interpretation, panel (b) presents the *R* value map under the optimal analytical condition (Table 3), and panel (c) displays the *R* value map under the most common analytical configuration applied uniformly across all sites (Table 4). Figure 6, Figure 7, Figure 8, Figure 9, Figure 10, Figure 11, Figure 12, Figure 13 and Figure 14 show that high *R* value clusters were generally concentrated in areas corresponding to archaeologically identified features. In some cases, high *R* values were also observed in surrounding natural soils, which aligns with the false-positive classifications shown in Table 3 (approximately 25% to 40% of natural soils exhibited *R* ≥ 1). Nevertheless, for most sites, the spatial concentration of high *R* values corresponded well with the central portions of the identified archaeological features.

Across all sites, balanced accuracy (BA) ranged from 0.70 to 0.86. Most sites exhibited balanced accuracy between 0.70 and 0.79 (sites T1–T5), while some sites achieved values above 0.80 (sites T2 and T6–T9). Sites with higher balanced accuracy generally showed larger mean *R* values for archaeological soils, such as 5.83 for site T6 and 4.00 for site T8. In contrast, sites with lower balanced accuracy tended to have mean *R* values closer to the threshold value of 1.

For archaeological soils, more than 80% of spectra exhibited *R* values greater than 1 for most sites. In addition, approximately 60% of natural soils had *R* values below 1, indicating that a proportion of natural soils (approximately 40%) exceeded the threshold under certain conditions.

Effect sizes (Cohen’s d) exceeded 1.0 for all sites, indicating substantial separation between archaeological and natural soil *R* value distributions. Similarly, Mann–Whitney U tests yielded *p*-values below 0.01 for all sites, confirming that archaeological and natural soil groups were significantly different.

Overall, the proposed PCA-based approach produced statistically significant and practically meaningful differentiation between archaeological and natural soils across all sites. Although the result depended on various site conditions, balanced accuracy values above 0.7, effect sizes greater than 1, and significant Mann–Whitney results indicated clear and consistent spectral differentiation during the feature identification process during excavation.

#### 3.2.2. Site-Level Observation

Site T1 (Joseon Dynasty residential site) achieved a balanced accuracy of 0.7506 under the optimal condition of 400–1000 nm wavelength range, regional N_soil_, raw spectra, and PC1-based reconstruction. Under this condition, 87.69% of archaeological soils showed *R* ≥ 1, while 62.42% of natural soils showed *R* < 1. As shown in Figure 6b, large *R* values were primarily concentrated within the upper portion of the grid, which corresponds to the archaeological feature identified through traditional feature identification. However, localised high *R* values were also observed in natural soils on the right side of the grid, just below the archaeological feature.

Site T2 represents a natural feature that was misinterpreted as a corridor tomb during field identification. When the site was evaluated under the optimal analytical setting (400–2400 nm, local N_soil_, continuum removal, PC1–3), the dataset resulted in a balanced accuracy of 0.8333. Figure 7b shows spatial differentiation between the area initially interpreted as archaeological and the surrounding natural soils. Although this site does not contain archaeological remains, the spectral differentiation pattern suggests that localised soil variation may produce deviations relative to surrounding natural soils.

The remaining sites (sites T3–T9) also demonstrated consistent discrimination performance under site-specific optimal conditions.

Site T3 (corridor tomb) achieved a balanced accuracy of 0.7901 using the 1000–2400 nm range with regional N_soil_, SNV preprocessing, and PC1-based reconstruction, with 91.67% of archaeological soils showing *R* ≥ 1. In Figure 8b, although several elevated *R* values were present in surrounding natural soils, most of the high *R* values are concentrated within the area corresponding to the boundary of the tomb feature.

Sites T4 and T5 (residential contexts) yielded balanced accuracy values of 0.7066 and 0.7205, respectively, under different combinations of wavelength range and N_soil_ definition, indicating moderate but stable separation performance. Figure 9 and Figure 10 show the concentration of elevated *R* values within the central portions of the archaeological features, while portions of the surrounding natural soils exhibit similar *R* values.

Higher classification performance was observed for sites T6–T9. In particular, site T6 (semi-subterranean house) achieved the highest balanced accuracy (0.8615) under 400–1000 nm, local N_soil_, and PC1–3 configuration. Sites T7–T9 (pit features) exhibited balanced accuracy values above 0.80 under various wavelength and preprocessing combinations.

Across all sites, highly balanced accuracy values were generally linked with stronger spatial clustering of high *R* values in the central parts of the archaeological features.

### 3.3. Influence of Analytical Parameters

#### 3.3.1. Spectral Preprocessing

**Figure 15:**
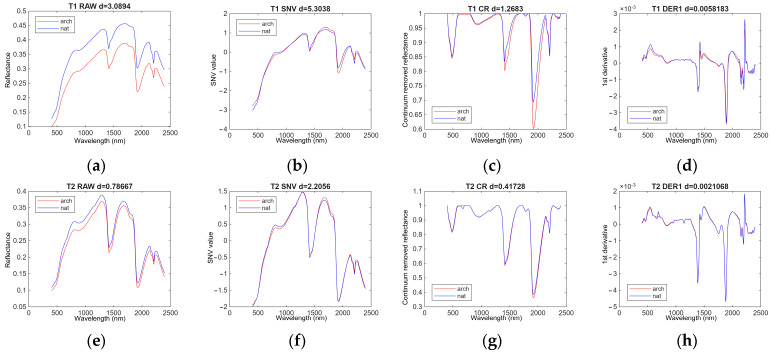
Mean raw and preprocessed soil spectral profiles for sites T1 (archaeological feature) and T2 (natural feature) under different preprocessing pipelines. The Euclidean distance (*D* value) between the mean archaeological and mean natural soil spectra is also indicated. The x-axis represents wavelength (nm), while the y-axis represents reflectance for the raw spectra, SNV-transformed values for the SNV spectra, continuum-removed values for the CR spectra, and first-derivative values for the DER1 spectra. Archaeological soils are shown in red and natural soils in blue. (**a**–**d**) Spectra for site T1: (**a**) raw reflectance; (**b**) standard normal variate (SNV); (**c**) continuum removal (CR); (**d**) first derivative (DER1). (**e**–**h**) Spectra for site T2: (**e**) raw reflectance; (**f**) SNV; (**g**) CR; (**h**) DER1.

Figure 15 illustrates the soil spectra for sites T1 and T2 within the 400–2400 nm range under different preprocessing conditions to highlight the differences in spectral features between the mean archaeological and natural soils. The Euclidean distance between the two spectra is also presented in the figure to demonstrate the differences between archaeological and natural spectra. The preprocessing methods used in this study (SNV, CR, and DER1) mathematically transform the original reflectance spectra, which may cause the resulting values to extend beyond the original reflectance range. In this figure, the top row images represent a spectral dataset from a typical archaeological site (site T1) and the bottom row represents the non-archaeological site (site T2). As described earlier, site T2 is a natural soil site that was misinterpreted as an archaeological site during field identification. For site T1, the raw spectra and SNV-processed spectra showed larger *D* values (3.0894 and 5.3038). In addition, site T2 showed smaller *D* values than site T1, illustrating that the misinterpreted archaeological soils were spectrally closer to natural soils. Higher *D* values in raw and SNV preprocessing provided an initial indication that these preprocessing approaches may produce better discrimination.

As shown in Table 3, raw spectra were frequently selected as part of the optimal configurations for several sites (sites T1, T6, T7, and T9), showing balanced accuracy values between 0.75 and 0.86. CR was optimal for sites T4 and T8 (BA = 0.7066 and 0.8333, respectively). SNV preprocessing showed optimal performance for sites T3 and T5 (BA = 0.7901 and 0.7205, respectively). However, DER1 preprocessing was not consistently selected among high-performing configurations.

Overall, no single preprocessing method consistently dominated across all sites, indicating that the effectiveness of preprocessing is site-dependent. However, raw reflectance and continuum removal appeared most frequently among high-performing configurations.

#### 3.3.2. Wavelength Range

Among the four primary windows, the 400–1000 nm wavelength range most frequently produced strong classification performance across multiple sites (sites T1, T5, T6, and T8), with balanced accuracy values consistently above 0.75 and exceeding 0.80 in some cases. The NIR range alone (1000–2400 or 1000–1800 nm) also produced competitive performance in certain contexts. For example, site T3 achieved a balanced accuracy of 0.7901 using 1000–2400 nm, while site T9 reached 0.8077 under the 1000–1800 nm range. In contrast, the visible range alone (400–700 nm) was optimal only for site T7, where a balanced accuracy of 0.8333 was achieved. Sub-range analysis also revealed site-specific optimisation patterns. The 700–1000 nm window was optimal for site T4, while the 1000–1800 nm window provided the highest performance for site T9 (BA = 0.8077).

Overall, no single wavelength range consistently outperformed others across all sites. However, the repeated selection of the 400–1000 nm window indicated stable performance.

#### 3.3.3. N_soil_ Definition

Local N_soil_ most frequently appeared with high classification accuracy, particularly for sites T6–T9, where balanced accuracy exceeded 0.80. Regional N_soil_ showed competitive results in certain contexts (sites T3–T5), with balanced accuracy values around 0.75. Global N_soil_ was optimal only for site T4 (BA = 0.7066), which showed the lowest performance among all sites.

Across all sites, configurations using local N_soil_ were consistently among the highest-performing combinations.

#### 3.3.4. PC Set Selection

For sites T1, T3, and T5, the optimal configuration involved using PC1 alone, resulting in balanced accuracy values between 0.72 and 0.79. In contrast, inclusion of higher-order components (PC1–3) improved the performance for sites T4 and T6–9. The highest balanced accuracy observed in the dataset (0.8615 for site T6) was achieved using PC1–3.

The results indicate that PC1 captures the dominant spectral variance, but inclusion of additional higher PCs also improved classification performance in several cases.

### 3.4. Standardised Conditions Across Sites

Based on the parameter sensitivity analysis presented in the previous sections, the wavelength range of 400–1000 nm and the use of local N_soil_ were identified as the most recurrent components among high-performing configurations, forming the standardised condition. To evaluate performance stability under the standardised configuration, these two parameters were fixed, while preprocessing and PC selection were allowed to vary in order to determine the best achievable performance within this constrained setting.

Table 4 summarises the balanced accuracy obtained for each site under the 400–1000 nm and local N_soil_ conditions. Across all sites, balanced accuracy ranged from 0.6390 (site T4) to 0.8615 (site T6). Excluding site T2, which represents a natural feature, the mean balanced accuracy was 0.7426. In comparison, the mean balanced accuracy of optimal conditions was 0.7879 (based on Table 3). Although performance decreased relative to the optimal configurations, most sites maintained balanced accuracy values above 0.70 under the standardised setting.

The corresponding spatial distributions are presented in section (c) of Figure 6, Figure 7, Figure 8, Figure 9, Figure 10, Figure 11, Figure 12, Figure 13 and Figure 14. Compared to the optimal conditions shown in section (b) of Figure 6, Figure 7, Figure 8, Figure 9, Figure 10, Figure 11, Figure 12, Figure 13 and Figure 14, the standardised conditions (400–1000 nm with local N_soil_ setting) generally produced lower *R* values and less sharply defined boundaries between archaeological and natural soils. Nevertheless, high *R* values remained spatially concentrated within areas corresponding to the archaeological features.

Overall, although the optimal condition gave the highest performance, the standardised conditions (400–1000 nm with local N_soil_) still produced stable discrimination across most sites, indicating practical applicability of the method to various archaeological excavations.

## 4. Discussion

The objective of this study was not to develop a predictive model transferable across sites, but to evaluate whether spectral deviation within each archaeological site can distinguish archaeological materials relative to natural soil (N_soil_). Therefore, each spectrum was evaluated independently, without partitioning the data into training and test sets. Performance metrics, such as balanced accuracy, were used to quantify the separability under a fixed threshold (*R* ≥ 1). The *R* value represents the degree of spectral deviation from the reference natural soil dataset (N_soil_). Higher *R* values indicate greater divergence from the background soil spectral signature, which may reflect alterations caused by anthropogenic activities such as soil mixing, organic matter accumulation, burning, or structural disturbance. In this context, the *R* value does not directly identify specific archaeological materials, but rather highlights areas where soil properties differ from the local natural baseline. Accordingly, it serves as a threshold to quantify the separability of archaeological materials.

### 4.1. Parameter Sensitivity

#### 4.1.1. Spectral Preprocessing

Soil reflectance spectra generally reflect overall brightness (albedo) as well as characteristic absorption features related to soil composition. Broad absorption features around 1400 nm and 1900 nm are commonly associated with water content, while features near 2200 nm are typically related to clay minerals. In the visible region (400–700 nm), variations in reflectance are often influenced by iron oxides and organic matter. Although these features are present in both archaeological and natural soils (Figure 3), subtle differences can be observed in their intensity and spectral shape. For example, variations in absorption depth around 1900 nm and 2200 nm, as well as differences in reflectance levels in the visible region, may reflect changes in moisture conditions, mineral composition, and anthropogenic inputs. In addition, as shown in Figure 3, the natural soil spectra exhibit a more distinct shoulder-like feature in the 800–900 nm region, which may be related to Fe-related VIS–NIR transitions [83,84], suggesting differences in iron-related properties between archaeological and natural soils.

These spectral characteristics are further emphasised depending on the preprocessing method applied (Figure 15). SNV reduces albedo effects and enhances relative absorption features, making bands around 1400 nm, 1900 nm, and 2200 nm more pronounced. CR further isolates individual absorption features, particularly around 1900 nm, 2200 nm, and 2330 nm, allowing clearer comparison of absorption band depth, position, and shape between archaeological and natural soils. In contrast, DER1 enhances local spectral variation but also amplifies noise under field conditions.

These differences in preprocessing influence the effectiveness of the proposed method across sites. Raw reflectance spectra were frequently selected under optimal conditions (sites T1, T6, T7, and T9). The effectiveness of raw spectra suggests that differences in overall reflectance intensity between archaeological and natural soils are substantial. Anthropogenic activities such as burning, organic matter accumulation, compaction, or mixing can alter soil colour and albedo, resulting in brightness shifts that are detectable in raw spectra. Therefore, preserving absolute reflectance information appears advantageous for distinguishing archaeological features from surrounding soils.

CR also showed strong performance for two sites (sites T4 and T8). Unlike raw reflectance, CR suppresses overall brightness and emphasises absorption features and spectral shape, which are more closely related to soil composition. This indicates that archaeological disturbance may modify both surface appearance and internal soil composition.

SNV preprocessing yielded optimal performance for some sites (sites T3 and T5), indicating that reducing scatter-related variability may improve the discrimination of archaeological features from background soil.

In contrast, DER1 did not perform well in this study. Although DER1 enhances absorption features, it can also amplify noise under field conditions.

#### 4.1.2. Wavelength Range

Extending soil spectral observations into the near-infrared region enables a more objective and quantitative framework for detecting subtle soil alteration patterns beyond the visible range. Multiple wavelength ranges were evaluated in this study to assess their relative effectiveness under field conditions. Among them, the 400–1000 nm wavelength range demonstrated the most consistent performance across multiple sites, suggesting that this spectral window captures key discriminative information under field conditions. This range includes not only the visible region, which is strongly influenced by soil colour and iron oxide content, but also the shortwave near-infrared region (700–1000 nm), where mineralogical information is present.

However, two sites (sites T3 and T9) exhibited optimal performance within near-infrared ranges (1000–1800 or 1000–2400 nm). This indicates that discrimination between archaeological and natural soils is not determined solely by colour differences. Absorption features, clay content, or moisture conditions in the near-infrared region may become more influential at sites where colour contrast between archaeological and natural soils is limited. These results imply that anthropogenic activities affect both soil colour and mineralogical composition.

These findings suggest that archaeological soil signatures in the VIS-NIR spectra are not governed by a single factor but are instead influenced by multiple physical and chemical changes resulting from human activity.

#### 4.1.3. N_soil_ Definition

In this study, local N_soil_ most commonly appeared as the optimal condition. This is because local N_soil_ captures the immediate background variability surrounding each archaeological feature and, therefore, defines a more precise natural soil spectral pattern for the study site. This enhances the sensitivity of the proposed PCA-based reconstruction, allowing anthropogenic spectral features to be distinguished from typical natural soil spectral characteristics.

Regional N_soil_ also produced good results for three sites (sites T1, T3, and T5). This indicates that broader background variability can still provide a stable reference under certain conditions. However, performance was generally slightly lower than that achieved with local N_soil_. This suggests that increasing the heterogeneity of the reference dataset may reduce sensitivity.

Global N_soil_, derived from an external spectral library, showed limited performance in the present dataset. Although global libraries offer standardised and widely accessible reference spectra, they introduce additional variability related to geographic diversity and sampling conditions. Furthermore, spectral resampling procedures may reduce the comparability between field-acquired and library spectra. These factors can weaken the detection of archaeologically relevant spectral features.

Overall, the results demonstrate that the effectiveness of the proposed PCA-based classification depends strongly on how background soil variability is defined. Calibration using locally acquired natural soil spectra provided the most stable performance within this dataset.

#### 4.1.4. PC Set Selection

The influence of PC selection further clarifies the nature of archaeological spectral variation. While PC1 explained most of the spectral variance across all sites, configurations including higher-order components (PC1–3) performed better in several cases across the nine study sites. This suggests that, although the dominant background variability is captured by PC1, archaeological disturbance may also be influenced by higher-order spectral variations.

Overall, these findings indicate that the identification of archaeological soil involves various factors such as reflectance intensity, soil composition, reference background soil, and higher-order PCs. The contribution of these factors varies by site, reflecting differences in feature type, depositional history, and environmental conditions. Therefore, this study shows that archaeological spectral signatures are influenced by multiple mechanisms.

### 4.2. Statistical Discrimination and Spatial Coherence

The overall result showed moderate to high balanced accuracy and large effect sizes, indicating that the method can separate archaeologically influenced soils from surrounding natural soils. Effect sizes exceeded 1.0 across all sites, whereas balanced accuracy values varied between sites. This reflects the fact that Cohen’s d measures separation between group averages, whereas balanced accuracy depends on how many samples fall on either side of the fixed threshold (*R* ≥ 1).

The relationship between quantitative classification metrics and spatial patterns is a critical aspect of the proposed workflow. Highly balanced accuracy values were generally associated with spatially structured distributions of high *R* values in 2D maps (Figure 6, Figure 7, Figure 8, Figure 9, Figure 10, Figure 11, Figure 12, Figure 13 and Figure 14). For most sites, high *R* values formed clusters that spatially coincided with archaeologically identified feature zones, rather than appearing randomly across the excavation area.

Although false positives were observed for several sites, these were typically concentrated around the archaeological features and did not form separate clusters. At site T1, a secondary concentration of high *R* values was observed downslope (right side of the image) from the primary feature zone. Since this site was located on a hillside, this pattern may reflect post-depositional movement or redistribution of anthropogenically influenced soils.

The clearest example of boundary delineation was observed at site T3. Here, the tomb structure exhibited relatively well-defined high *R* values. At other sites, boundaries were less sharply expressed. However, this does not necessarily indicate a limitation of the method. Archaeological features, particularly residential areas and mixed-use contexts, often involve soil disturbance, mixing, and gradual transitions. Consequently, partial boundary diffusion in spectral maps may reflect inherent archaeological processes rather than analytical limitations.

In addition, several measurements (sites T6–T9) were conducted along transects crossing only a part of the archaeological feature because of time constraints. Despite this limited spatial coverage, elevated *R* values were still observed within the archaeological feature zones, suggesting that the method may also be applicable at sites with limited measurement coverage.

Importantly, spatial distributions derived from the proposed PCA-based method allow archaeologists to visually and quantitatively interpret excavation surfaces during feature identification. The integration of statistical discrimination metrics (balanced accuracy and effect size) with spatial clustering of *R* values demonstrates that the method provides both quantitative validation and spatial interpretation. Thus, combined statistical–spatial results support the practical applicability of the proposed approach.

The spatial distribution of *R* values shows variation across sites and may also be related to differences in archaeological feature types. For example, the tomb feature at site T3 exhibits a relatively confined concentration of high *R* values, corresponding to a well-defined burial context. In contrast, several residential sites (sites T1, T4–T6) display more spatially distributed or heterogeneous patterns, which may reflect more extensive soil disturbance and mixing associated with habitation activities. Pit features (sites T4, T7, T8) tend to show localised but distinct clusters, suggesting more discrete depositional or disturbance events. Although these observations are based on a limited number of sites and variable measurement conditions, they suggest that spatial patterns of spectral deviation may reflect differences in archaeological context. This highlights the potential of the proposed method to capture archaeologically meaningful variability across different feature types.

### 4.3. Implications for Rescue Excavation

The primary objective of this study was to develop a rapid, quantitative, and reproducible workflow for feature identification during rescue excavation. The results demonstrate that VIS-NIR spectroscopy, combined with the proposed PCA-based framework, can provide immediate surface-level discrimination between archaeologically influenced soils and surrounding natural backgrounds once the topsoil has been removed.

From a practical perspective, a handheld spectrometer covering the 400–1000 nm wavelength range appears sufficient for stable performance in most tested sites. Under excavation conditions, natural soil spectra collected around the site can be used to define a local reference (N_soil_), enabling on-site calibration without reliance on laboratory processing. In situations where reliable local natural soil references cannot be defined during fieldwork, regional or global reference datasets may provide primary baselines. However, in this study, local reference spectra produced the most stable discrimination performance. Therefore, systematic collection of background soil spectra during early stages of excavation is recommended to maximise analytical sensitivity.

The proposed workflow does not require complex spectral inversion or advanced machine-learning models. Instead, it relies on PCA-based analysis, which is computationally efficient and can be applied using relatively simple data processing. This makes the approach suitable for time-constrained rescue archaeology, where rapid decision-making is essential.

Importantly, the integration of quantitative metrics with spatial *R* value mapping provides archaeologists with both numerical and visual outputs. Such outputs can be integrated into existing excavation practices and may improve the accuracy, efficiency and reproducibility of feature identification.

Overall, this study suggests that field-based spectroscopy can serve as a practical decision-support tool during excavation, reducing subjective uncertainty and enhancing the reproducibility of feature identification.

### 4.4. Limitations and Future Work

Several limitations should be acknowledged in the present study. Because the method was designed for time-efficient application during rescue excavation, spectral measurements were conducted under practical field constraints. As a result, sample sizes varied among sites, grid configurations were not fully standardised, and in some cases (sites T6–T9), measurements were collected only along transects rather than across complete feature surfaces. While this reflects realistic excavation conditions, it may limit the direct comparability of spatial patterns across sites. Nevertheless, even with limited spectral measurement, the method was able to highlight regions with a high probability of archaeological material, as shown for sites T6–T9. Accordingly, the results should be interpreted within the context of site-specific conditions, and the proposed approach is not intended as a universally transferable model but rather as a framework sensitive to local soil variability.

Additionally, although PC1 explained more than 90% of total spectral variance at all sites, the inclusion of higher-order components (PC2–PC3) often improved classification performance. These components account for only a small proportion of total variance but may capture subtle spectral variations related to anthropogenic disturbance. Although principal component analysis (PCA) can, in some cases, be linked to specific soil properties, the aim of this study is not to attribute individual principal components to particular physicochemical variables. Instead, PCA is used as a statistical tool to capture overall spectral variability and to enhance the detection of relative differences between archaeological and natural soils. Further investigation is required to determine which specific soil properties, such as mineral alteration, organic matter enrichment, moisture variation, or compaction effects, contribute to these higher-order spectral signals. Such analysis may provide additional insights and is suggested as a direction for future research.

An additional limitation of this study is the difference in scale between the spectrometer sampling area and the grid resolution used for spatial interpretation. The TerraSpec Halo Mineral Identifier spectrometer samples a small surface area relative to the size of each grid cell, and therefore, a single measurement may not fully capture within-cell soil heterogeneity. This mismatch may introduce local variability in *R* values, particularly in contexts where soil properties change over small spatial scales. However, the proposed workflow is primarily intended to evaluate relative spectral deviation and broader spatial clustering patterns, rather than to treat individual point measurements as complete representations of entire grid cells. Therefore, results should be interpreted with greater emphasis on overall spatial trends than on fine-scale local variation. Future studies may benefit from the use of spectrometers with a larger sampling area, allowing a greater surface area to be sampled at each measurement location.

Overall, the results indicate that the method identifies statistical differences from defined natural datasets (N_soil_) rather than directly detecting archaeological features. This suggests that the workflow is better interpreted as an anomaly detection approach rather than as a strict archaeological classifier. Accordingly, the observed spectral contrasts may reflect not only primary depositional differences but also secondary pedogenic and post-depositional processes, as shown at site T2.

The present dataset is limited to a specific geographic region and primarily includes archaeological features from the Joseon period (with one Bronze Age site). The results of this study should be interpreted within this contextual scope, and caution is required when extending the findings to other archaeological or environmental settings. Therefore, expanding the analysis to a wider range of archaeological feature types, soil backgrounds, and cultural periods would allow a more comprehensive evaluation of the proposed workflow under diverse conditions.

Future work should therefore focus on applying the method under more controlled conditions across a broader range of environmental and archaeological contexts, as well as geographic regions. In addition, integrating spectral analysis with mineralogical investigation could help clarify the origins of the observed spectral variations, particularly those influenced by higher-order PCs. Future studies should also explore integration with geophysical or geochemical techniques to further enhance the reliability of archaeological feature detection.

## 5. Conclusions

This study proposes and systematically evaluates a quantitative workflow for archaeological feature identification during excavation using field-based VIS-NIR spectroscopy combined with a PCA-based spectral deviation approach. The results demonstrate that archaeological soils can be effectively differentiated from surrounding natural soils.

Among the tested parameters, the 400–1000 nm wavelength range and locally defined N_soil_ configurations yielded the most stable performance within the dataset. Across nine study sites, balanced accuracy values exceeded 0.70 under optimised conditions, with several sites achieving values above 0.80. Strong statistical performance was accompanied by spatial clustering of high *R* values within archaeologically identified feature zones. This consistency between quantitative metrics and spatial patterns supports the practical interpretability of the method during excavation.

An important implication of this study is that archaeological signatures are best interpreted relative to local natural soil variability rather than a universal background reference. Accordingly, the proposed approach evaluates deviations from locally defined soil conditions instead of relying on absolute spectral signatures.

Overall, the proposed spectrometer-based workflow provides an objective supplement to traditional feature identification processes. By integrating quantitative spectral analysis with spatial anomaly mapping, the method enhances reproducibility and reduces subjective uncertainty in archaeological excavation. These findings demonstrate that field spectroscopy has strong potential as a decision-support tool in excavation practice. Furthermore, although this study focused on field-based, excavation-stage application, the methodological framework may be extended to broader contexts, including bare-soil airborne remote sensing for detecting archaeological features at larger spatial scales.

## Figures and Tables

**Table 1:** Summary of the nine study sites (sites T1–T9).

Site	Feature Type	Period	Feature Size (m)	Grid Size (m)	Total Spectra	Archaeological Spectra	Natural Spectra
T1	Residential site	Joseon Dynasty	1.2 × 2.1	0.1 × 0.1	222	65	157
T2	Natural feature	-	2.4 × 1.8	0.3 × 0.3	48	30	18
T3	Corridor tomb (ring-ditch tomb)	Joseon Dynasty	1.8 × 3.4	0.2 × 0.2	152	48	104
T4	Residential site, corridor tomb, and pit	Joseon Dynasty	4.5 × 4.0	0.25 × 0.5	105	49	56
T5	Residential site	Joseon Dynasty	4.5 × 4.5	0.25 × 0.25	544	305	239
T6	Semi-subterranean house	Joseon Dynasty	0.5 × 4.5	0.25 × 0.5	49	37	12
T7	Pit	Bronze Age	0.5 × 4.0	0.5 × 05	24	9	15
T8	Pit	Joseon Dynasty	0.5 × 3.5	0.5 × 0.5	21	9	12
T9	Pit	Joseon Dynasty	0.5 × 4.0	0.5 × 0.5	25	12	13

**Table 3:** Result for best site conditions.

Site	Wavelength	N_soil_	Preprocessing	PCs	Balanced Accuracy	Mean of *R_a_*	% of *R_a_* ≥ 1	% of *R_n_* < 1	Effect Size	Mann–Whitney U Test
T1	400–1000	Regional	RAW	PC1	0.7506	1.07	87.69	62.42	1.3549	6.85681 × 10^−14^
T2	400–2400	Local	CR	PC1-3	0.8333	1.77	100.00	66.67	1.3064	0.0000314
T3	1000–2400	Regional	SNV	PC1	0.7901	1.25	91.67	66.35	1.0179	3.76936 × 10^−13^
T4	700–1000	Global	CR	PC1-3	0.7066	1.04	87.76	53.57	1.4207	0.0000068
T5	400–1000	Regional	SNV	PC1	0.7205	1.51	84.26	59.83	1.0871	6.14932 × 10^−24^
T6	400–1000	Local	RAW	PC1-3	0.8615	5.83	97.30	75.00	1.3570	0.0000117
T7	400–700	Local	RAW	PC1-3	0.8333	3.11	100.00	66.67	2.3258	0.0000647
T8	400–1000	Local	CR	PC1-3	0.8333	4.00	100.00	66.67	2.0836	0.0015643
T9	1000–1800	Local	RAW	PC1-3	0.8077	3.09	100.00	61.54	1.4174	0.0042952

**Table 4:** Results for local N_soil_ at 400–1000 nm wavelength range.

Site	Preprocessing	PCs	Balanced Accuracy	Mean of *R_a_*	% of *R_a_* ≥ 1	% of *R_n_* < 1	Effect Size	Mann–Whitney U Test
T1	SNV	PC1-2	0.7057	2.37	80.00	61.15	0.8536	SNV
T2	DER1	PC1	0.7611	1.67	96.67	55.56	1.5416	DER1
T3	CR	PC1-3	0.6490	1.31	75.00	54.81	0.6234	CR
T4	RAW	PC1-2	0.6390	1.24	65.31	62.50	0.3992	RAW
T5	RAW	PC1-2	0.6893	1.63	78.03	59.83	0.9117	RAW
T6	RAW	PC1-3	0.8615	5.83	97.30	75.00	1.3570	RAW
T7	SNV	PC1	0.8000	2.16	100.00	60.00	1.9963	SNV
T8	CR	PC1-3	0.8333	4.00	100.00	66.67	2.0836	CR
T9	SNV	PC1-2	0.7628	4.29	83.33	69.23	1.5597	SNV

## Data Availability

The raw data supporting the conclusions of this article will be made available by the author on request.
